# Examining the Role of Social Determinants of Health in Preventable Hospitalization Rates in Miami-Dade County, Florida

**DOI:** 10.7759/cureus.105499

**Published:** 2026-03-19

**Authors:** Kayla L Haydon, Joel J Mantilla, Sakhi Shah, Chelsey Ameda, Ann-Katrin Valencia, Nana Aisha Garba

**Affiliations:** 1 Department of Humanities, Health, and Society, Florida International University Herbert Wertheim College of Medicine (FIU HWCOM), Miami, USA

**Keywords:** ambulatory care-sensitive conditions, health disparities, hospitalization rates, primary care, social determinants of health

## Abstract

Introduction

Ambulatory care-sensitive conditions (ACSCs) are acute or chronic health issues with increased preventable hospitalization rates. Literature shows that elevated ACSC hospitalization rates are associated with social determinants of health (SDoH), limited healthcare access, and poor health outcomes. The objective of this study was to compare SDoH and hospitalization rates in two adjacent municipalities in Miami-Dade County, Florida.

Methods

Time trends of ACSC hospitalization rates from 2017 to 2021 in Florida, Miami-Dade County, & Miami Gardens were obtained utilizing established ACSC diagnoses: diabetes and associated complications, heart failure, hypertension, adult asthma, and chronic obstructive pulmonary disease (COPD). A secondary comparison of ACSC hospitalization rates and social determinants of health (SDoH) with the geographically adjacent municipality, Miami Lakes, was conducted.

Results

ACSC hospitalizations in Miami Gardens were double the hospitalizations in Miami-Dade County and Florida overall (e.g., 85.4 [78.7-92.1] per 10,000 in Miami Gardens vs. 34.6 [34.2-35.0] per 10,000 in Miami-Dade County vs. 35.8 per 10,000 in Florida due to heart failure). However, the hospitalization rates in Miami Lakes were much lower. Descriptive analysis of SDoH between both cities indicated significant disparities, with a greater percentage of households in Miami Gardens living below the federal poverty level, receiving SNAP, and lacking Internet access.

Conclusion

Preventable ACSC hospitalizations in Miami Gardens, FL, have remained high in recent years, in comparison to the state of Florida and Miami-Dade County. A secondary descriptive comparison with the geographically adjacent municipality, Miami Lakes, reveals that these elevated hospitalization rates may be associated with SDoH. Further research should continue to explore trends in ACSC hospitalization rates within underserved communities in the US to uncover possible health disparities.

## Introduction

Preventable hospitalization rates are used to study the quality and performance of primary care in a community [1]. A subset of conditions with high preventable hospitalization rates is known as ambulatory care-sensitive conditions (ACSCs). These conditions are thought to be effectively addressed by ambulatory or outpatient care through preventative medicine, early disease detection and management, and high continuity of care [2]. A large cross-sectional study of Medicare claims data revealed that greater access to primary care decreases the probability of ACSC hospitalizations [3]. ACSCs include diabetes, hypertension, cardiovascular disease, and asthma [1,4].

While national rates for ACSC hospitalizations have not been consistently reported, previous literature has determined a significant annual reduction following Medicaid expansion in participating states [5]. Additionally, there is evidence that social determinants of health (SDoH) are associated with elevated ACSC hospitalization rates [4]. According to the World Health Organization, SDoH are factors that affect health found where people are born, grow, live, work, and age. The five pillars of SDoH are health and healthcare, social and community context, neighborhood and built environment, economic stability, and education [6]. SDoH associated with increased risk for ACSCs include food security, exercise, smoking, alcohol consumption, exposure to stressors, financial stability, and education [7,8]. Moreover, patients living in rural areas have decreased quality of ambulatory care for chronic conditions and preventative screening rates [9]. Increased barriers to outpatient care access, such as lack of health insurance and provider availability, have resulted in higher preventable hospitalization rates, particularly in Hispanic populations [10]. In rural areas in the United States, decreased access to care and variation in severity of disease have led to 40% higher preventable hospitalization and emergency department visit rates and 23% higher mortality rates from ACSCs overall [9]. Healthy People 2030 is focused on reducing preventable hospitalizations to improve the health and well-being of populations. Preventable hospitalizations are also a significant area of concern because they contribute to a large portion of healthcare spending in the United States [4]. This study aims to understand the role of SDoH in elevated hospitalization rates in Miami Gardens, Florida, as compared to an adjacent municipality, Miami Lakes, Florida.

## Materials and methods

This is an ecological time-trend analysis of ACSC hospitalization rates from 2017 to 2021 in Florida, Miami-Dade County, and two adjacent municipalities within Miami-Dade County (Miami Gardens and Miami Lakes).

Data was obtained from publicly available databases from the Miami-Dade Matters website. This data is reported by licensed healthcare facilities to the Florida Agency for Health Care Administration. Three-year rolling age-adjusted hospitalization rates per 10,000 adults for ACSC categories (diabetes overall, short-term and long-term complications of diabetes, uncontrolled diabetes, type 2 diabetes, heart failure, hypertension, adult asthma, and COPD) were obtained. ACSC definitions align with the Agency for Healthcare Research and Quality (AHRQ) Prevention Quality Indicators (PQIs) [11]. We performed descriptive analyses of age-adjusted ACSC hospitalization rates to account for differences in healthcare utilization and mortality rates in different-aged populations [12].

Indicators of SDoH, including health insurance coverage, internet subscription, vehicle availability, SNAP participation, income and poverty, educational attainment, linguistic isolation, and homeownership, were also obtained from the Miami-Dade Matters website, sourced from the American Community Survey. We performed descriptive analyses of the SDoH indicators in Miami Gardens and Miami Lakes to contextualize patterns in ACSC hospitalization rates. All analyses were conducted using Microsoft Excel (Redmond, USA). 

## Results

From 2017 to 2021, there was a decrease in ACSC hospitalization rates in Miami Gardens, Miami-Dade County, and the State of Florida (Table 1). For example, the hospitalization rate due to heart failure in Miami Gardens was 90.2 (83.3-97.1) per 10,000 from 2017 to 2019 and decreased to 85.4 (78.7-92.1) per 10,000 from 2019 to 2021. Miami-Dade County generally had lower hospitalization rates than Florida overall. From 2019 to 2021, the hospitalization rate due to heart failure in Miami-Dade County was 34.6 (34.2-35.0) per 10,000. However, Miami Gardens' hospitalization rates (85.4 [78.7-92.1] per 10,000) were double those of Miami-Dade County and Florida (35.8 per 10,000).

**Table 1:** Age-adjusted (18+ years) three-year rolling hospitalization Rates (per 10,000) in Miami Gardens (Zip Code 33054), Miami-Dade County, and Florida from 2017 to 2021 from the Florida Agency for Healthcare Administration (MiamiDadeMatters.Org). Confidence interval reported if available. Diabetes: Primary diagnoses of type 1 & 2 diabetes. Gestational diabetes excluded Long-term complications of diabetes: Cases include a primary diagnosis of a long-term complication of diabetes. Long-term complications of diabetes include eye, renal, neurological, or circulatory complications or complications not otherwise specified. Cases of gestational diabetes are excluded. Short-term complications of diabetes: Cases include a primary diagnosis of a short-term complication of diabetes. Short-term complications of diabetes include ketoacidosis, hyperosmolarity, or coma. Cases of gestational diabetes are excluded. Type 2 Diabetes: Only cases where the first-listed diagnosis is type 2 diabetes are included. Uncontrolled diabetes: Cases include a primary diagnosis of uncontrolled diabetes. Cases of gestational diabetes, cases with mention of short-term complications, and cases with mention of long-term complications are excluded. Heart Failure: Cases with a cardiac procedure are excluded. Hypertension: Cases with kidney disease combined with a dialysis access procedure and cases with a cardiac procedure are excluded. Adult asthma: Asthma cases with a secondary diagnosis of cystic fibrosis or other respiratory anomalies are excluded. COPD: COPD cases with a secondary diagnosis of cystic fibrosis or other respiratory anomalies are excluded.

	2019-2021			2018-2020			2017-2019		
	Miami-Gardens	Miami-Dade County	Florida	Miami-Gardens	Miami-Dade County	Florida	Miami-Gardens	Miami-Dade County	Florida
Diabetes	51.2 (45.9-56.5)	22 (21.7-22.3)	27.1	52.9 (47.5-58.3)	22.3 (22.0-22.6)	27.4	56.5 (50.9-62.1)	23.2 (22.8-23.6)	27.1
Long-term complications of diabetes	24.2 (20.6-27.8)	12 (11.8-12.2)	12.4	26.4 (22.6-30.2)	12.1 (11.9-12.3)	12.4	28.6 (24.6-32.6)	12.8 (12.5-13.1)	12.5
Short-term complications of diabetes	18.2 (15.0-21.4)	5.9 (5.7-6.1)	9.3	16.6 (13.5-19.7)	5.9 (5.7-6.1)	9.5	15.9 (12.9-18.9)	5.4 (5.2-5.6)	8.7
Type 2 Diabetes	38.1 (33.6-42.6)	17.7 (17.4-18.0)	20.3	39.3 (34.7-43.9)	18.0 (17.7-18.3)	20.5	43.1 (38.2-48.0)	18.7 (18.4-19.0)	19.9
Uncontrolled diabetes	8.8 (6.6-11.0)	4 (3.9-4.1)	5.3	9.7 (7.4-12.0)	4.3 (4.2-4.4)	5.5	11.9 (9.4-14.4)	4.7 (4.9-5.1)	5.8
Heart Failure	85.4 (78.7-92.1)	34.6 (34.2-35.0)	35.8	84.4 (77.8-91.0)	36.0 (35.6-36.4)	36.4	90.2 (83.3-97.1)	39.3 (38.9-39.7)	37.9
Hypertension	23.6 (20.2-27.2)	8 (7.8-8.2)	8.3	26.6 (22.8-30.4)	8.3 (8.1-8.5)	8.4	28.7 (24.7-32.7)	9.1 (8.9-9.3)	8.8
Adult asthma	6.6 (4.7-8.5)	3.4 (3.3-3.5)	3.7	8.2 (6.0-10.4)	4.1 (3.9-4.3)	4.4	12.6 (9.9-15.3)	4.8 (4.6-5.0)	5.1
COPD	23.7 (20.2-27.2)	10.5 (10.3-10.7)	14.6	27.7 (23.9-31.5)	14.1 (13.8-14.4)	18.3	39.4 (34.8-44.0)	20.2 (19.2-20.5)	24.7

Miami Gardens vs. Miami Lakes: a comparison of SDoH

We then performed a secondary comparison of the same ACSC hospitalization rates in the adjacent municipality within Miami-Dade County, Miami Lakes, due to its geographic proximity. This observational analysis revealed that Miami Lakes’ hospitalization rates were lower than those in Miami Gardens. ACSC hospitalization rates in Miami Lakes were lower than hospitalization rates in Miami-Dade County, while ACSC hospitalization rates in Miami Gardens were double the county hospitalization rates (Table 2).

**Table 2:** Age-Adjusted (18+ years) Three-Year Rolling Hospitalization Rates (per 10,000) in Miami Gardens (Zip Code 33054) and Miami Lakes (Zip Code 33018) from 2019-2021 from the Florida Agency for Healthcare Administration (MiamiDadeMatters.Org). Confidence interval reported if available. Diabetes: Primary diagnoses of type 1 & 2 diabetes. Gestational diabetes excluded. Long-term complications (LTC) of diabetes: Cases include a primary diagnosis of a long-term complication of diabetes. Long-term complications of diabetes include eye, renal, neurological, or circulatory complications or complications not otherwise specified. Cases of gestational diabetes are excluded. Short-term complications (STC) of diabetes: Cases include a primary diagnosis of a short-term complication of diabetes. Short-term complications of diabetes include ketoacidosis, hyperosmolarity, or coma. Cases of gestational diabetes are excluded. Type 2 Diabetes: Only cases where the first-listed diagnosis is type 2 diabetes are included. Uncontrolled diabetes: Cases include a primary diagnosis of uncontrolled diabetes. Cases of gestational diabetes, cases with mention of short-term complications, and cases with mention of long-term complications are excluded. Heart Failure: Cases with a cardiac procedure are excluded. Hypertension: Cases with kidney disease combined with a dialysis access procedure and cases with a cardiac procedure are excluded. Adult asthma: Asthma cases with a secondary diagnosis of cystic fibrosis or other respiratory anomalies are excluded. COPD: COPD cases with a secondary diagnosis of cystic fibrosis or other respiratory anomalies are excluded.

ACSC Condition​	Miami Gardens	Miami Lakes
Diabetes ​	51.2 (45.9-56.5)​	17.1 (14.9-19.3)​
LTC of diabetes​	24.2 (20.6-27.8)​	9.1 (7.6-10.6)​
STC of diabetes ​	18.2 (15.0-21.4)​	5.2 (3.9-6.5)​
Type 2 Diabetes ​	38.1 (33.6-42.6)​	12.4 (10.6-14.2)​
Uncontrolled Diabetes	8.8 (6.6-11.0)​	2.7 (1.9-3.5)​
Heart Failure ​	85.4 (78.7-92.1)​	29.9 (27.2-32.6)​
Hypertension ​	23.6 (20.2-27.2)​	6.2 (4.9-7.5)​
Adult asthma ​	6.6 (4.7-8.5)​	1.9 (1.2-2.6)​
COPD​	23.7 (20.2-27.2)​	6.0 (4.8-7.2)​

An evaluation of SDoH between both cities (Table 3) revealed that residents of Miami Gardens were generally younger in age and identified as Black or African American and Latino. Households in Miami Gardens were less likely to have health insurance, Internet access, or a personal vehicle. Residents of Miami Gardens reported a much lower median household income ($51,067) compared to residents in Miami Lakes ($84,504). Renters in Miami Gardens were more likely to spend more than 30% of their income on rent. A greater percentage of households in Miami Gardens also lived below the federal poverty level and received SNAP with children. Residents of Miami Gardens were less likely to hold a high school diploma and/or bachelor’s degree by the age of 25 and were also less likely to be employed. A more comprehensive comparison of SDoH between the two municipalities is included in Table 3.

**Table 3:** Community Characteristics of Miami Gardens (Zip Code 33054) and Miami Lakes (Zip Code 33018) from 2019-2021 from the Florida Agency for Healthcare Administration (MiamiDadeMatters.Org).

​	Miami Gardens-33054​	Miami Lakes-33018​
Population by Age​	​	​
<18​	26.33%​	19.85%​
18+​	73.67%​	80.15%​
25+​	63.71%​	71.76%​
65+​	14.32%​	15.39%​
85+​	1.83%​	1.86%​
Average Household Size​	3.05 persons​	3.26 persons​
Race​	​	​
White​	14.69%​	25.01%​
Black/African American​	50.31%​	1.53%​
American Indian/Alaskan Native​	0.21%​	0.15%​
Asian​	0.34%​	1.18%​
Native Hawaiian/Pacific Islander​	0.04%​	0.02%​
Other​	10.87%​	14.28%​
2+ races​	23.55%​	57.84%​
Ethnicity​	​	​
Hispanic/Latino​	47.61%​	92.31%​
Non-Hispanic/Latino​	52.39%​	7.69%​
Social Determinants​	​	​
Adults without Health Insurance​	29.30%​	28.90%​
Linguistic isolation ​	10.70%​	14.10%​
Households with an Internet subscription​	77.80%​	94.70%​
Households without a Vehicle​	8.70%​	3.60%​
Households receiving SNAP with Children​	54.10%​	30.10%​
Households with Cash Public Assistance Income​	10.10%​	2.00%​
Homeownership​	61.40%​	60.60%​
Renters spending 30% of more of household income on rent ​	71.80%​	50.80%​
Median household income​	$51,067 ​	$84,504 ​
Households living below poverty​	16.90%​	6%​
People living 200% above poverty level​	55.20%​	78.80%​
People 25+ with a bachelor’s degree or higher​	16.30%​	39.20%​
People 25+ with a high school diploma or higher​	81%​	91.30%​

## Discussion

Many of the U.S. Department of Health’s Healthy People 2030 national objectives focus on reducing preventable hospital visits due to chronic conditions [13]. Our cross-sectional comparison of two municipalities within Miami-Dade County in Florida revealed that hospitalizations due to ACSCs may be associated with SDoH.

Across multiple ACSC categories, Miami Gardens exhibited persistently higher age-adjusted hospitalization rates than Miami-Dade County and Florida, with especially large gaps relative to the adjacent municipality of Miami Lakes. These disparities were observed across a range of ACSCs, including diabetes, heart failure, hypertension, chronic obstructive pulmonary disease (COPD), and asthma. Differences in hospitalization rates between Miami Gardens and Miami Lakes were greatest due to diabetes and heart failure. These findings are consistent with the role of ACSC hospitalizations as a sentinel indicator of primary care access and quality as operationalized in the AHRQ quality indicators [11].

Our work comparing two adjacent municipalities supports previous research revealing substantial geographic variation in ACSC hospitalizations not fully explained by clinical need alone. The persistent elevation in hospitalization rates in Miami Gardens appears to correlate with multiple unfavorable SDoH indicators, including higher poverty rates, lower levels of educational attainment, reduced internet access, greater reliance on public assistance, and limited access to private transportation. Transportation barriers have been reported to account for deferred or missed healthcare for millions of adults in the US and disproportionately affect lower-income communities [14,15]. This is especially significant considering the importance of regular and timely follow-up for chronic conditions. Limited internet access has also been associated with decreased access to timely ambulatory care [16]. The demographic profile of Miami Gardens further underscores the intersection of race, ethnicity, and socioeconomic status in shaping health outcomes, as a majority of residents identify as Black or African American and non-Hispanic. This reflects structural and social inequities rather than biological differences that lead to increased barriers to healthcare and health disparities [17]. Other studies particularly discuss significant between-area differences in hospitalization rates related to healthcare access, lack of continuity of care, and lack of local resources [18-20].

Community-based interventions to address ACSC hospitalizations include employing community health workers to connect residents with long-term medical and social support services, increasing access to primary care services, and promoting continuity of care within the community [2,21]. Programs such as the Green Family Foundation Neighborhood Health Education Learning Program (Neighborhood HELP) at Herbert Wertheim College of Medicine have successfully employed multidisciplinary teams in providing household-centered care and addressing social risk factors [22]. Ultimately, reducing preventable hospitalizations and improving the population's well-being cannot be done without considering the cultural and socioeconomic factors affecting the health of said populations.

Limitations of this study include reliance on publicly available, aggregate data for our analysis, limiting the ability to control confounders at the individual level, such as health behaviors, comorbidities, and healthcare utilization patterns. Additionally, hospitalization rates are derived from diagnostic coding practices, which may be inconsistent between hospitals and over time, potentially introducing misclassification bias. Another limitation is that SDoH indicators were only assessed at the zip code level and may not fully capture neighborhood-specific nuances. It is also important to consider that the cross-sectional and ecological nature of our study restricts the ability to analyze causal relationships between SDoH and hospitalization rates, especially on the individual level. Lastly, since our study was a comparison of two municipalities in Florida, further research should use a much larger sample size, analyzing many regions across the US, to fully understand the impact of SDoH on hospitalization rates.

Future studies should aim to integrate more individual-level data on both health outcomes and SDoH indicators to better assess causality. Longitudinal cohort studies tracking residents over time could provide insight into how changes in SDoH influence hospitalization trends. Additionally, qualitative research involving community members and healthcare providers could offer valuable perspectives on perceived barriers to care and enhance the development of appropriate interventions. Evaluating the impact of ongoing policy initiatives, such as Medicaid expansion or federally qualified health center (FQHC) programs, on ACSC hospitalization rates within underserved communities would further strengthen the evidence base for targeted public health strategies.

## Conclusions

Preventable ACSC hospitalizations in Miami Gardens, FL, have remained high in recent years, in comparison to the state of Florida and Miami-Dade County. Secondary comparison with the geographically adjacent municipality, Miami Lakes, reveals that these elevated hospitalization rates may be associated with SDoH such as transportation, income, and education. This study reinforces the need for comprehensive public health efforts that address the structural and social inequities underlying disparities in preventable healthcare utilization. Continued focus on community-tailored interventions and policy reforms is essential to reduce preventable hospitalizations and promote health equity in underserved urban communities. Further research should continue to explore trends in ACSC hospitalization rates within underserved communities in the United States to promote health equity. 
